# Epoxy Resin Highly Loaded with an Ionic Liquid: Morphology, Rheology, and Thermophysical Properties

**DOI:** 10.3390/gels11120992

**Published:** 2025-12-10

**Authors:** Svetlana O. Ilyina, Irina Y. Gorbunova, Michael L. Kerber, Sergey O. Ilyin

**Affiliations:** 1 A.V. Topchiev Institute of Petrochemical Synthesis, Russian Academy of Sciences, 29 Leninsky Prospect, 119991 Moscow, Russia; 2 Department of Plastics Processing Technology, D. Mendeleev University of Chemical Technology of Russia, 9 Miusskaya Square, 125047 Moscow, Russia

**Keywords:** epoxy resin, ionic liquid, curing, rheology, thermophysical properties

## Abstract

An epoxy resin can be crosslinked with an imidazole-based ionic liquid (IL), whose excess, provided its high melting temperature, can potentially form a dispersed phase to store thermal energy and produce a phase-change material (PCM). This work investigates the crosslinking of diglycidyl ether of bisphenol A (DGEBA) using 1-ethyl-3-methylimidazolium chloride ([EMIM]Cl) at its mass fractions of 5, 10, 20, 40, and 60%. The effect of [EMIM]Cl on the viscosity, curing rate, and curing degree was studied, and the thermophysical properties and morphology of the resulting crosslinked epoxy polymer were investigated. During the curing, [EMIM]Cl changes its role from a crosslinking agent (an initiator of homopolymerization) and a diluent of the epoxy resin to a plasticizer of the cured epoxy polymer and a dispersed phase-change agent. An increase in the [EMIM]Cl content accelerates the curing firstly because of the growth in the number of reaction centers, and then the curing slows down because of the action of the IL as a diluent, which reduces the concentration of reacting substances. In addition, a rise in the proportion of [EMIM]Cl led to the predominance of the initiation over the chain growth, causing the formation of short non-crosslinked molecules. The IL content of 5% allowed for curing the epoxy resin and elevating the stiffness of the crosslinked product by almost 7 times compared to tetraethylenetriamine as a usual aliphatic amine hardener (6.95 GPa versus 1.1 GPa). The [EMIM]Cl content of 20–40% resulted in a thermoplastic epoxy polymer capable of flowing and molding at elevated temperatures. The formation of IL emulsion in the epoxy matrix occurred at 60% [EMIM]Cl, but its hygroscopicity and absorption of water from surrounding air reduced the crystallinity of dispersed [EMIM]Cl, not allowing for an effective phase-change material to be obtained.

## 1. Introduction

Epoxy polymers are thermosetting materials whose gel network is formed by the reaction of epoxy resins with hardeners [1,2], which determine their wide range of applications [3,4,5,6,7,8,9,10]. Their advantages are high strength and stiffness, good adhesion, chemical resistance, and electrical insulation, while disadvantages include brittleness, exothermic curing, and the toxicity of many hardeners [11,12,13]. The choice of hardener depends on the required curing time and temperature, as well as the operational temperatures and mechanical properties of the cured product [14]. According to the type of reactive group, hardeners can be anhydrides, thiols, amines, and various nitrogen-containing compounds [15,16,17], such as imidazoles, which are used as hardeners or can act as curing accelerators with other hardeners [18,19,20,21].

Like other imidazoles, imidazole-based ionic liquids can potentially interact with epoxy groups to cure epoxy resins [22,23,24,25]. However, their non-volatility aligns with the principles of green chemistry, giving them an advantage over other organic hardeners [26,27,28]. The effectiveness of ILs as hardeners depends on their miscibility with the epoxy resin, viscosity, and ability to interact with epoxy groups [29]. The application of ILs may impart new functionalities to epoxy materials, enhance their physicochemical properties, and eliminate the need for highly toxic conventional hardeners [22,30,31].

There are several unique advantages of ILs as hardeners.

First, ILs have high ionic conductivity, which makes them promising components for batteries, fuel cells, electrochromic devices, capacitors, sensors, and separation membranes [32,33,34,35,36,37]. However, pure ILs are liquids and can flow out of devices. For this reason, it is rational to incorporate the ILs into a polymer matrix, e.g., based on epoxy resin, to provide shape stability. Acting as conductive plasticizers or hardeners, ILs have the potential to impart an ionic conductivity to epoxy polymers.

Second, nitrogen-containing anions show bactericidal properties [38,39,40,41,42], which can be used to potentially create antimicrobial coatings and composites [43,44], medical instruments [45,46], and food packaging films [47,48] resistant to bacteria and fungi growth. Ionic composites may also be beneficial in shipbuilding for producing ship hulls resistant to fouling by algae and other marine organisms [49,50,51,52]. A key aspect is the incorporation of ionic fragments into the polymer structure [53], which prevents their leaching into the environment and the gradual reduction in the effectiveness of the bactericidal material. ILs acting as curing agents can chemically incorporate into the epoxy polymer network [54], thereby minimizing the negative environmental impact of the resulting material.

Third, high-melting ILs may serve as components of phase-change materials (PCMs), which can store or release significant amounts of heat during phase transitions, making them useful for thermal energy storage [55,56,57]. PCMs can be used in solar energy storage systems [58,59,60], for regulating indoor temperatures [61,62], and in batteries and electronic components of computing devices to prevent overheating [63,64,65]. However, a common issue with many PCMs is the loss of their shape at elevated temperatures, which can be overcome by creating composite PCMs. The epoxy polymer can act as a matrix for form-stable PCMs, incorporating dispersed crystallizing organic compounds [66,67,68]. A high-melting IL used in excess can simultaneously act as (1) a curing agent for the epoxy resin and (2) a dispersed phase-change agent in the final composite material, potentially suitable as a thermal energy accumulator.

Thus, ILs can play four roles for epoxy resins: as a non-toxic curing agent, an ion-conductive additive, a bactericide, and a phase-change agent for creating antibacterial coatings, ion-exchange membranes, thermal energy accumulators, and other materials without requiring an additional crosslinking agent. When acting as a curing agent, the IL will integrate into the three-dimensional network of the epoxy polymer, potentially imparting antibacterial and ion-exchange properties. However, in the case of PCMs, the IL must have a high melting temperature and be in excess relative to the epoxy resin for supplemental acting as a dispersed phase. Moreover, the higher the content of the crystallizing dispersed phase is, the greater the heat storage capacity of the resultant PCM. In other words, the epoxy resin should be cured using the maximum possible amount of the IL to create a highly filled form-stable composite.

Despite the wide range of possible additional functions of ILs, no studies have yet been conducted to obtain crosslinked epoxy polymers containing different IL mass fractions—from a low concentration for curing to a high concentration aimed at producing a PCM. This work aims to study the effect of IL concentration on the rheology, structure, and thermophysical properties of the epoxy resin before and after curing. 1-Ethyl-3-methylimidazolium chloride ([EMIM]Cl) serves as the model IL because of its ability to cure epoxy resins [69] and its relatively high melting temperature of 80 °C, which will allow it to crystallize at a high concentration within the crosslinked epoxy polymer to produce a PCM. Thus, the novelty of this work lies in the first systematic investigation of an imidazolium-based IL, specifically [EMIM]Cl, across a broad concentration range (5–60 wt%) within a chemically cured epoxy network. This approach enables the IL to transition from a crosslinker to a plasticizer, and ultimately to a dispersed phase-forming agent—a functional regime previously unexplored and essential for evaluating the feasibility of IL-based phase-change materials. These findings provide not only fundamental insights into structure–property relationships in IL/epoxy systems, but also practical design rules for selecting IL content depending on the target application (e.g., high-stiffness thermoset vs. moldable thermoplastic vs. PCM).

## 2. Results and Discussion

### 2.1. Uncured Epoxy Resin/IL Mixtures

**Figure 1:**
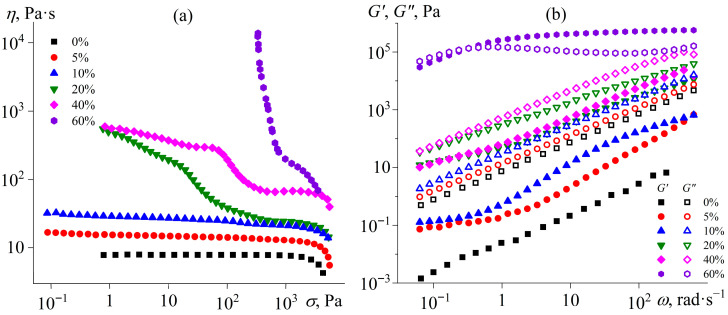
Dependences of viscosity on shear stress (**a**) and storage and loss moduli on angular frequency (**b**) for the epoxy resin containing [EMIM]Cl at 25 °C. The mass fraction of the ionic liquid is indicated in the legend.

**Figure 2:**
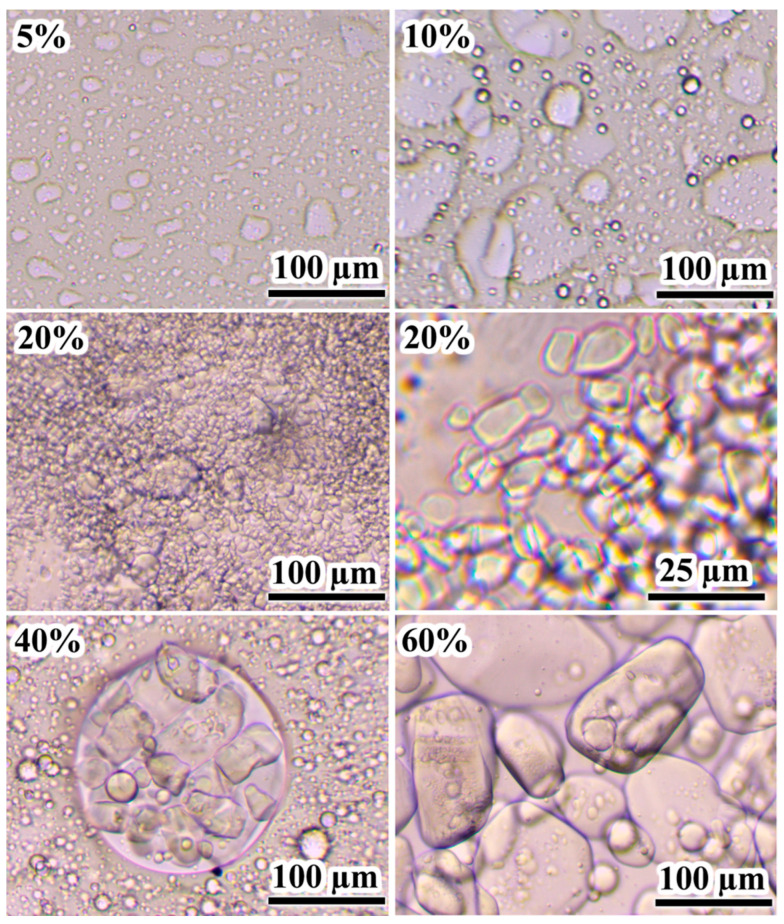
Microphotographs of uncured epoxy mixtures containing [EMIM]Cl, the concentration of which is indicated in weight percentages.

The flow curves of uncured epoxy mixtures at 25 °C demonstrate the effect of the IL on their rheological properties. Since the melting point of [EMIM]Cl is approximately 80 °C, this IL in the epoxy resin can be in a dispersed crystalline state, affecting the effective viscosity. Pure epoxy resin exhibits Newtonian behavior up to a shear stress of about 2 kPa, after which its effective viscosity slightly decreases (Figure 1a). When 5–10% [EMIM]Cl is added, Newtonian behavior is retained across a wide range of shear stresses, although the effective viscosity gradually increases with a rise in the IL concentration. The increase in viscosity may be attributed to both (1) the dissolution of the IL within the epoxy resin and (2) the formation of an emulsion whose dispersed droplets likely represent a saturated solution of the epoxy resin within the IL (Figure 2, 5–10%). Although these droplets are optically resolvable and significantly exceed colloidal dimensions, their volume fraction remains sufficiently low to prevent both percolated network formation and shear-induced structuring. Consequently, the systems exhibit behavior characteristic of dilute emulsions, wherein the dispersed phase does not experience strong flow-mediated interactions. This results in near-constant viscosity across a broad range of applied shear stresses.

A further increase in the IL content to 20% enhances the non-Newtonian behavior of the mixtures, raising the effective viscosity at low shear stresses (Figure 1a). According to optical microscopy data, this IL concentration leads to the appearance of dispersed crystals in the epoxy medium, with sizes ranging from 5 to 10 µm (Figure 2, 20%). An increase in the IL content to 40% causes a noticeable rise in effective viscosity at medium and high shear stresses while maintaining flowability. The crystals’ size increases to 25–50 µm, and they gather into a liquid dispersed phase consisting of IL saturated with epoxy resin (Figure 2, 40%). In the case of 60% [EMIM]Cl, a pronounced yield stress appears at 340 Pa, which can result from the appearance of a spatial structural network of IL crystalline particles contacting each other because of their high concentration (Figure 2, 60%).

The dependences of the storage and loss moduli on angular frequency confirm the formation of the structural network from the IL phase (Figure 1b). An increase in the IL concentration in the epoxy resin, which serves as a liquid-like reference sample for comparison, allows tracking the evolution of viscoelastic properties. As the IL is added, the dynamic moduli gradually increase, likely due to the emergence of an interface between droplets from the IL (more accurately, from a saturated solution of epoxy resin in the IL) and the epoxy resin medium (a saturated solution of the IL in the resin). During periodic deformation, the IL droplets tend to restore their spherical shape, increasing the overall elasticity of the colloid system. At IL contents exceeding 20%, dispersed crystals appear, which can agglomerate to form a spatial network [70]. However, no structuring of the mixtures occurs at the IL concentrations of 5–40%. For these systems, the loss modulus exceeds the storage modulus across the entire frequency range, and both moduli decrease as the angular frequency lowers. This fact indicates that these mixtures retain liquid-like behavior. The situation changes upon adding 60% IL into the epoxy resin. In this case, the dynamic moduli significantly increase, and the storage modulus exceeds the loss modulus at high frequencies, likely due to the formation of a structural network from IL crystalline particles.

### 2.2. Curing of Epoxy Resin/IL Mixtures

**Figure 3:**
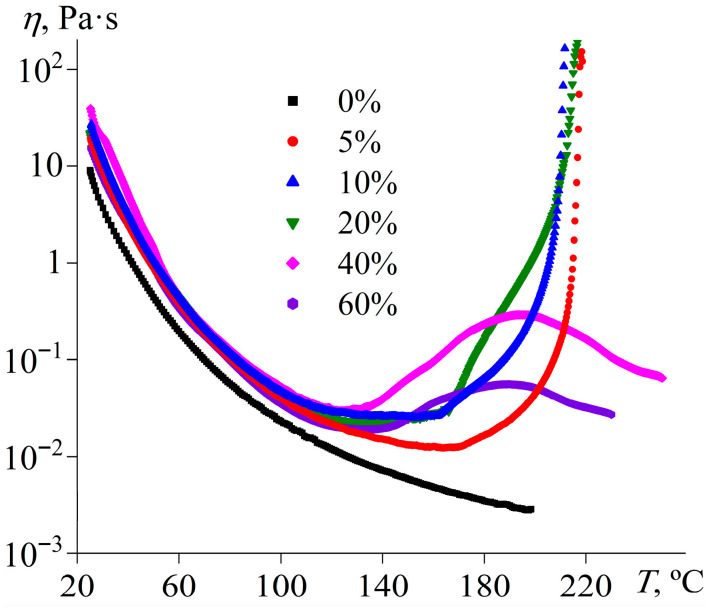
Temperature dependencies of effective viscosity at a shear rate of 100 s^−1^ for the epoxy resin containing [EMIM]Cl, whose mass fraction is indicated in the legend.

Because of the ability of [EMIM]Cl to act as a curing agent for epoxy resins, it is possible to determine the effect of its concentration on the curing rate and completeness. The effective viscosity of pure epoxy resin at 25 °C is lower than that of mixtures containing the IL because of the absence of a dispersed phase (Figure 3). Upon heating, the viscosity of the resin gradually decreases without any signs of curing due to the lack of a hardener. The effective viscosity of IL-containing mixtures is higher than that of the pure resin and comparable for all considered IL concentrations, but only at low temperatures before the curing starts. The systems containing 5–20% IL show the most intense crosslinking, which manifests via a sharp increase in viscosity at heating due to rapid growth in polymer molecular weight, culminating in a three-dimensional network of covalent bonds [71]. The temperature at which viscosity starts rising decreases with an increase in the IL content from 164 °C (5% [EMIM]Cl) to 130 °C (20% [EMIM]Cl), while the loss of flowability occurs at comparable temperatures around 212–218 °C.

**Figure 4:**
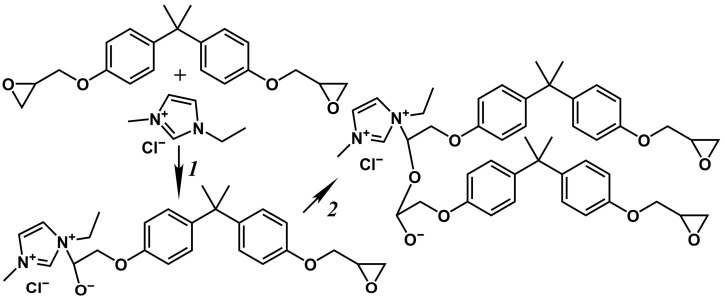
Scheme of the chemical interactions between the epoxy resin and [EMIM]Cl: an initiation (*1*) and homopolymerization (*2*).

In the cases of 40% and 60% IL concentrations, the curing does not proceed completely, as the effective viscosity does not reach infinite values but passes through a local maximum and then decreases again (Figure 3). The observed change in the viscosity may indicate the epoxy resin polymerization without a resultant three-dimensional network of chemical bonds. In the initial stage of crosslinking, the IL acts as an initiator for the formation of reactive anions (Figure 4, reaction *1*), which polymerize in the second stage (reaction *2*). In this case, the IL catalyzes the homopolymerization of the epoxy resin. At low IL concentrations, few polymerization centers arise, leading to the creation of large crosslinked macromolecules. In contrast, many polymerization centers appear at high IL concentrations, and the epoxy resin molecules are consumed rapidly to form low-molecular-weight polymers. At 5–20% IL contents, there is an excess of epoxy groups relative to IL molecules by factors of 3.25–15.4 (Table 1), leading to a three-dimensional gel network. By contrast, epoxy groups are deficient at 40–60% IL concentrations, resulting in the predominant formation of linear, cyclic, or branched molecules capable of flowing.

**Figure 5:**
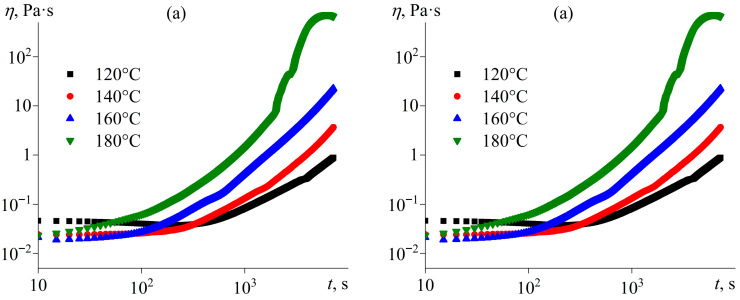
Dependencies of viscosity on curing time at a shear rate of 100 s^−1^ for (**a**) the epoxy resin containing 20% [EMIM]Cl at different temperatures and (**b**) the epoxy resin containing various concentrations of [EMIM]Cl at 160 °C.

The study of curing under isothermal conditions can help to establish the effects of temperature and IL concentration on the curing kinetics. Figure 5 shows examples of viscosity curves during isothermal curing. As expected, increased curing temperature accelerates crosslinking, leading to a more rapid rise in viscosity over time (Figure 5a). The influence of IL content is more complex (Figure 5b). An increase in the IL concentration from 5% to 20% accelerates crosslinking, which can be because of the higher content of the IL as a reactant, i.e., a higher number of initiation reactions (Figure 4, reaction *1*). However, a further increase in the IL concentration to 40% and then to 60% slows the viscosity growth, probably due to the role of the IL as a diluent, where its increased proportion reduces the concentration of reactive epoxy groups.

The high IL loadings of 40–60 wt% result in a non-monotonic evolution of viscosity with time, reflecting a transition from network-forming to non-network-forming polymerization dictated by the stoichiometric imbalance between epoxy groups and IL molecules. At lower IL concentrations (5–20 wt%), the excess of epoxy groups enables crosslinking of growing chains, leading to gel network formation and a divergence in viscosity due to gelation. In contrast, at IL loadings ≥ 40 wt%, the deficiency of epoxy groups (Table 1) shifts the reaction pathway predominantly toward chain termination and intramolecular cyclization, yielding low-molecular-weight oligomers. The initial rise in viscosity stems from rapid molecular weight growth in the early reaction stage, but its subsequent decrease indicates that the system remains in the liquid state, where the effective viscosity is governed by the concentration and size of the IL-soluble oligomers rather than by an elastic network.

The obtained rheokinetic curves allow for the rate of chemical reactions to be estimated. A challenge arises from the differing products of the curing reactions at IL contents of 5–20% and 40–60%. In the first case, a crosslinked polymer forms, while the second case yields low-molecular-weight linear, branched, and/or cyclic molecules. For this reason, it is impossible to describe the kinetics of crosslinking universally for all curing cases, leaving it possible to estimate the reaction rate at the initial stage, which is similar for all IL concentrations. The exponential equation can describe the initial parts of the viscosity curves [72]:

(1) $$\eta =\eta_{0}exp\left(k_{\eta }t\right)$$

where *η*_0_ is the initial viscosity, and *k_η_* is the kinetic constant, which increases at a higher reaction rate.

**Figure 6:**
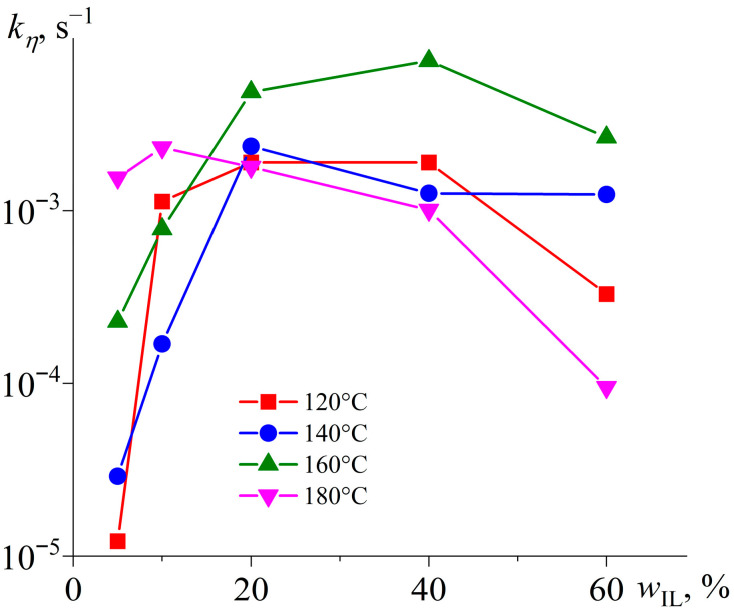
Dependencies of the kinetic constant of the first stage of epoxy resin curing on the weight fraction of [EMIM]Cl at different temperatures, indicated in the legend.

Figure 6 presents the results of determining the kinetic constant for various temperatures and IL contents. At an IL mass fraction of 5%, the rate of the initial crosslinking stage predictably increases with a temperature rise. Under these conditions, the IL acts as a reactant, and a subsequent increase in its content to 10–20% accelerates curing while reducing the role of temperature in determining the reaction rate. Nevertheless, further growth in the IL concentration slows the crosslinking as the IL starts acting as a diluent that reduces the concentration of reacting epoxy resin. Moreover, an elevation of temperature above 160 °C also slows down curing at high IL concentrations.

According to Figure 4, curing involves two stages. In the first stage, the IL initiates polymerization, while the second stage consists of the actual growth of polymer chains. Presumably, an increase in temperature intensifies the initiation more strongly than the chain growth. When there is a deficiency of the IL (*w*_IL_ = 5%), a temperature rise accelerates the overall crosslinking rate, as it increases the initiation rate, compensating for the lack of IL molecules as initiators. However, the excess IL causes the reaction of epoxy groups predominantly with the IL rather than with growing chains. This fact increases the concentration of reactive anionic groups, leading to their mutual reactions with the termination of chain growth and cyclization of molecules. In this case, heating to 180 °C accelerates the initiation, further suppressing the formation of high-molecular-weight products.

Therefore, the non-monotonic dependence of the kinetic constant *k_η_* on IL content (Figure 6) reflects a competition between chemical and diffusional control of the curing reaction. At low IL loadings (5–20 wt%), the reaction is chemically controlled: increasing the IL concentration raises the number of reactive anionic centers (Figure 4, reaction *1*), thereby accelerating chain initiation and early-stage propagation.

However, at *w*_IL_ ≥ 40 wt%, two diffusion-limiting effects become dominant:

(a) Dilution effect: the high concentration of IL reduces the effective molarity of the reactive epoxy groups, lowering the probability of intermolecular collisions necessary for chain growth (Figure 4, reaction *2*);

(b) Viscosity-mediated retardation: rapid polymerization during the early stage (Figure 5b) sharply increases the system’s viscosity, restricting molecular mobility and slowing down subsequent propagation, even at elevated temperatures.

The decrease in *k_η_* at 180 °C compared to 160 °C for the 60 wt% IL sample is particularly telling, suggesting that the higher temperature accelerates the initiation step so intensely that it results in a burst of short, non-gelling oligomers (as indicated by a lower Δ*H*_1+2_, discussed below). This rapid initial reaction depletes the epoxy groups before diffusion-limited chain extension can occur. In other words, the system transitions from growth-controlled to initiation-saturated kinetics, where an excess of reactive species suppresses network formation—a characteristic signature of stoichiometric imbalance in step-growth polymerizations.

Thus, the optimal IL concentration and temperature for rapid curing of the epoxy resin are 5% and 180 °C, respectively. However, 40–60% IL and 160 °C are best for producing a phase-change material with the highest polymerization rate. In the latter case, nevertheless, the formation of low-molecular-weight molecules may negatively affect the shape stability of the cured product.

**Figure 7:**
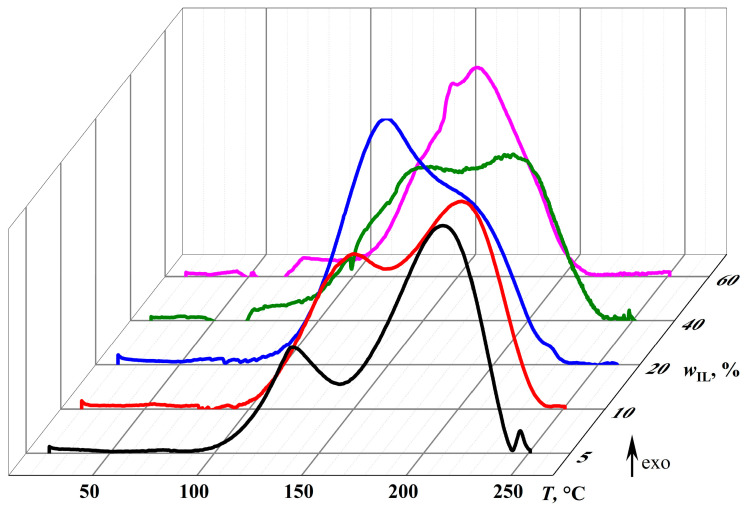
Changes in heat flow during the curing of the epoxy resin with different concentrations of [EMIM]Cl at a heating rate of 2 °C/min.

The calorimetric curves obtained during the curing of epoxy mixtures (Figure 7) confirm the change in the dominant chemical reactions upon altering the concentration of the IL, which acts as both an initiator and a diluent. At an IL content of 5%, the thermogram under gradual temperature rise has two asymmetric exothermic peaks at 140.0 °C and 210.6 °C (*T*_max,1_ and *T*_max,2_, Table 1). Presumably, the first smaller peak corresponds to the initiation reaction (Δ*H*_1_ = 99.3 J/g), while the second stands for the chain growth through epoxy homopolymerization (Δ*H*_2_ = 260.4 J/g).

Previously, two-stage curing featuring two exothermic peaks on DSC curves was reported for the DGEBA/1-butoxymethyl-1-methylimidazolium salicylate system at a 10/1 molar ratio. The authors attributed this behavior to the enhanced initiation properties of both the salicylate anion and the imidazolium cation [25]. However, at an equimolar DGEBA/IL ratio (1/1 mol/mol), the process transitioned to a single-stage curing with only one exothermic DSC peak. Furthermore, two-stage curing of DGEBA has been observed with 3–15 wt% 1-ethyl-3-methylimidazolium dicyanamide, which the authors associated with reactions between epoxy groups and cyanamide anions [73]. A similar effect was observed during curing with 0.99–8.3 wt% 1-butyl- or 1-decyl-3-methylimidazolium dicyanamides, but not with their tetrafluoroborate analogues, which was attributed to anionic polymerization of the epoxy resin initiated by thermal decomposition products of the 1,3-dialkylimidazolium ionic liquids [74]. Additionally, two-stage curing of DGEBA occurs with 3–25 mol% 2-ethyl-4-methylimidazole but not with 50 mol%. The proposed mechanism involves the reaction of the epoxy resin with the 1-unsubstituted imidazole in the first stage, forming an OH-containing adduct, followed by its etherification and polymerization in the second stage [75,76]. Therefore, in our system, the ionic nature of the curing agent also does not appear to be the determining factor for the observed two-stage process. The first exothermic peak likely corresponds to the reaction of epoxy groups with imidazole rings, while the second peak is associated with the subsequent polymerization step.

A rise in the IL content to 10% and then to 20% gradually increases the area of the first peak (to 223.3 and then to 333.9 J/g, respectively) and decreases the area of the second peak (to 205.6 and 158.4 J/g). This redistribution of thermal energy reflects an increase in the proportion of the initiation reaction and a corresponding reduction in the proportion of the chain growth reaction in the total curing thermal effect (Δ*H*_1+2_), which elevates from 359.3 J/g to 428.9–492.3 J/g.

A further increase in the IL concentration to 40% reduces Δ*H*_1+2_ and Δ*H*_1_ to 429.9 and 257.1 J/g, reflecting the decreasing content of epoxy groups relative to IL molecules, i.e., their deficiency in the total reactive mass. An even more significant rise in the IL content to 60% merges the exothermic initiation and chain growth peaks and further reduces the curing thermal effect to 327.5 J/g due to the diluent role of the IL and the decreased epoxy group proportion. The IL effect on the peak maximum temperatures is less pronounced. However, the decreased maximum temperature of the second exothermic peak is evident when the IL concentration rises from 5% to 20%, probably due to the increased number of growing chains and, as a result, their lower degree of polymerization and crosslinking.

Thus, the IL plays the role of both an initiator and a diluent, transiting predominantly from the first role to the second at about 40% mass fraction. At low IL concentrations, crosslinking proceeds with a more extensive thermal effect, i.e., more completely. In contrast, at high content of the IL, its part does not react with the epoxy resin, either forming a dispersed phase or remaining dissolved in the crosslinked epoxy polymer. Additionally, an increase in the IL proportion causes the formation of low-molecular-weight products instead of a crosslinked polymer because of the transition from a small reaction center number and the chain growth dominance to the initiation reaction prevalence and numerous reaction centers. All these factors should jointly affect the physicochemical properties of the resultant cured systems.

### 2.3. Cured Epoxy Resin/IL Mixtures

**Figure 8:**
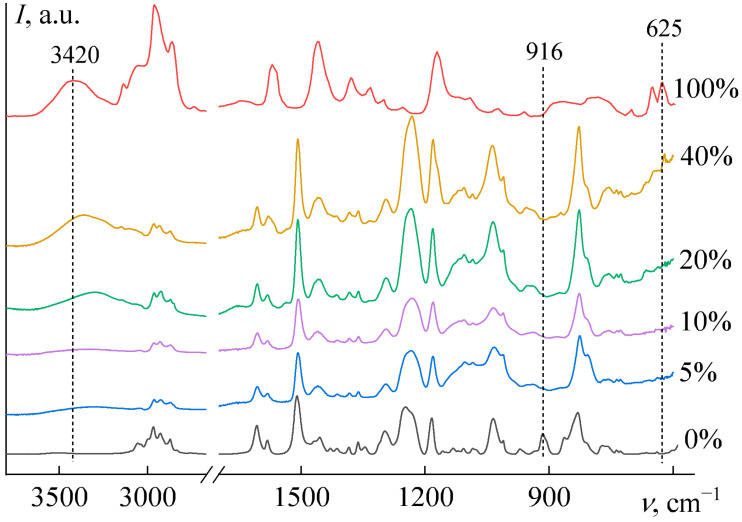
The FTIR spectra of epoxy resin, [EMIM]Cl, and their blends after curing, where the [EMIM]Cl weight fractions are indicated next to the spectra.

Analysis of the Fourier-transform infrared (FTIR) spectra (Figure 8) reveals that the absorption band at 916 cm^−1^, corresponding to epoxy groups [77], disappears completely after curing with as little as 5 wt% [EMIM]Cl, confirming the completeness of the reaction. With further increases in IL content, the epoxy groups continue to be fully consumed during curing.

A characteristic absorption band of the pure IL is observed at 625 cm^−1^ (Figure 8, 100% IL), which corresponds to deformation vibrations of the imidazole ring [78,79,80]. This band is absent in samples containing 5–20 wt% IL, indicating that the imidazole ring is incorporated into the epoxy network, suppressing these vibrations. In contrast, at 40 wt% IL, this band reappears in the spectrum, suggesting that a fraction of the IL remains unbound, existing in a dissolved or dispersed state.

Furthermore, the pure IL exhibits a broad band at 3420 cm^−1^, attributed to O–H stretching vibrations of free or weakly bound hydroxyl groups from absorbed water [81,82]. This band is absent in samples with 5–10 wt% IL, implying that absorbed water evaporates during high-temperature curing and that the cured material does not readily re-absorb moisture. Conversely, samples with 20 and 40 wt% IL display a hydroxyl absorption band, which is shifted to 3293 and 3362 cm^−1^, respectively. This shift indicates stronger and more cooperative hydrogen bonding [83,84], likely resulting from a lower overall water content and a transition from predominant water–water hydrogen bonds to a dominance of hydrogen bonds between water molecules and [EMIM]Cl ions. Moreover, the reabsorption of atmospheric moisture by the samples with *w*_IL_ ≥ 20 wt% after curing at 160 °C highlights the exceptional hygroscopicity of the ionic liquid. This observation is consistent with literature data showing that [EMIM]Cl can retain about 17 wt% water at 25 °C and 15% relative humidity [85].

**Figure 9:**
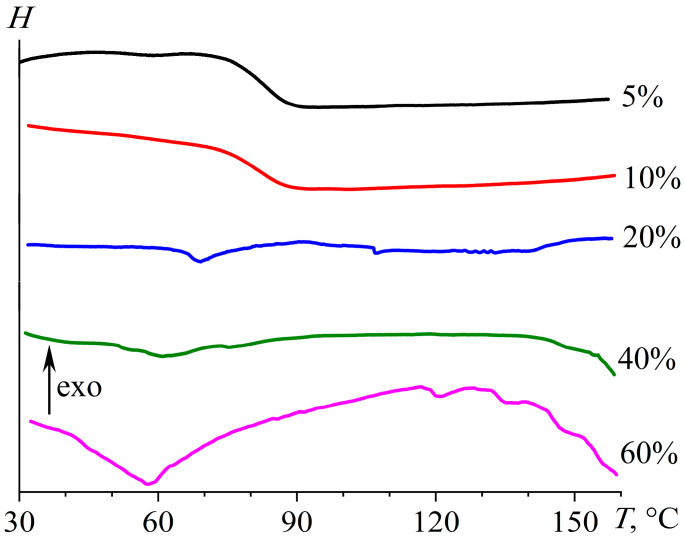
Heating thermograms of cured epoxy mixtures containing [EMIM]Cl, whose mass fraction is indicated near the curves.

The DSC thermogram of the epoxy polymer cured with 5% [EMIM]Cl exhibits a single glass transition at 83.3 °C with no signs of other thermal transitions (Figure 9). This fact indicates that the ionic liquid has fully reacted with the epoxy oligomer—otherwise, its melting endotherm, expected near 87.5 °C [86], would be observed. An increase in the IL mass fraction to 10%, 20%, and then 40% shifts the glass transition temperature to 81.3, 66.7, and 51.6 °C (Table 1), which may be because of a decrease in crosslink density and the action of excess IL as a plasticizer for the epoxy matrix. The highest IL concentration of 60% results in the appearance of an endothermic transition in the glass transition region with a minimum of 58.1 °C, likely due to the melting of dispersed ionic liquid. The melting enthalpy in this case is 3.34 J/g, which is too low to consider this material as a phase-change material. The melting enthalpy of pure [EMIM]Cl is 105.0 J/g [86], indicating a nominal crystallinity degree of 60% IL in the epoxy matrix as low as 5.3%. Such a low degree of crystallinity may be due to the high hygroscopicity of the IL, which absorbed water from the air even while embedded in the epoxy matrix. This hygroscopicity also reduces the IL melting point from 87.5 to 58.1 °C.

**Figure 10:**
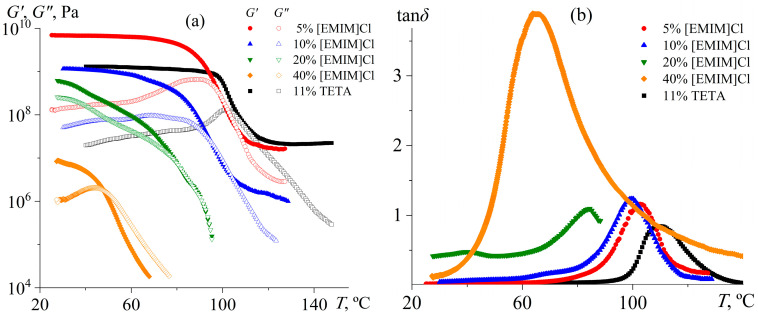
Temperature dependencies of storage and loss moduli (**a**) and loss tangent (**b**) for cured epoxy mixtures containing [EMIM]Cl, whose mass fraction is indicated in the legends. Data for the same epoxy resin cured with triethylenetetramine are shown for comparison.

The action of the IL as a plasticizer is evident during the dynamic mechanical analysis of the samples (Figure 10). Curing with 5% [EMIM]Cl yields a crosslinked epoxy polymer with a flexural modulus of 6.95 GPa and a glass transition temperature of 102.8 °C according to the loss tangent maximum. When compared, an epoxy polymer cured with an aliphatic amine as an ordinary curing agent (triethylenetetramine, TETA) exhibits a flexural modulus nearly seven times lower: 1.1 GPa (Table 2). Lower stiffness might suggest a lower crosslink density. However, the glass transition temperature of the TETA-cured epoxy polymer is higher (109.9 °C versus 102.8 °C), just like the higher its rubbery-state modulus (21.0 MPa versus 16.1 MPa).

In other words, TETA provides a higher crosslink density. Likely, the lower glass-state stiffness of the TETA-cured polymer is due to the incorporation of flexible ethylene fragments into the epoxy gel network if one compares it with the introduction of rigid imidazole rings from the IL. To test this hypothesis, we cured the epoxy resin at 180 °C using an aromatic amine—4,4′-diaminodiphenyl sulfone (DDS)—which contains no flexible aliphatic fragments. Its use results in a significantly higher glass transition temperature (175.3 °C, Table 2), indicating increased crosslink density and reduced segmental mobility compared to curing with [EMIM]Cl or TETA. The storage modulus in the glassy state (25 °C) for the DDS-cured resin is 2.96 GPa, nearly three times higher than that for the TETA-cured resin (1.10 GPa), yet still more than two times lower than for the [EMIM]Cl-cured resin (6.95 GPa). The superior stiffness of the [EMIM]Cl-cured system likely stems from the incorporation of the rigid, planar, and aromatic imidazolium ring into the polymer network, which restricts segmental motion more effectively than even the biphenyl-type linkages in DDS. The amine nitrogens in DDS allow for relatively free rotation around C–N bonds, whereas the tertiary nitrogen in the imidazolium ring is part of an aromatic system, resulting in restricted rotation and enhanced rigidity. In addition, DDS consists of two benzene rings connected via a sulfone group, permitting rotation of the rings relative to each other. In contrast, [EMIM]Cl contains a single imidazole ring, which reduces conformational freedom and promotes more compact crosslinks. For comparison, curing the same epoxy resin with methyltetrahydrophthalic anhydride (MTHPA) yields a much lower glass transition temperature (64.2 °C) and stiffness (1.06 GPa) [87] than curing with TETA, DDS, or [EMIM]Cl, despite the presence of an aromatic moiety. This result is likely due to the formation of flexible aliphatic segments (e.g., –CH_2_–CH(OH)–CH_2_–) between aromatic units, which increase chain mobility and reduce packing density.

An increase in the IL concentration to 10% decreases the flexural modulus of the epoxy polymer in both the glassy and rubbery states to 1.16 GPa and 1.5 MPa, respectively, indicating a reduction in crosslink density. This fact is also evident from the decrease in the glass transition temperature to 99.6 °C. The cured epoxy polymer retains its network structure since the storage modulus exceeds the loss modulus at high temperatures. The situation changes when the IL concentration rises to 20% and 40%: both moduli sharply decline, and the cured samples lose their shape stability upon heating above 60–80 °C. The emergence of their liquid-like behavior also results from the increase in the loss tangent maximum, especially at 40% IL content (Figure 10b). Thus, an IL concentration of about 20% causes a transition from crosslinked to thermoplastic polymers since the initiation reaction starts dominating over chain growth (Figure 4). In addition, an excess IL likely plasticizes the resulting thermoplastic polymer, implying its reduced viscosity and increased flowability. In this case, the absence of a rubbery plateau indicates that the resulting thermoplastic products are not high-molecular-weight polymers, but rather oligomers that lack macromolecular entanglements.

**Figure 11:**
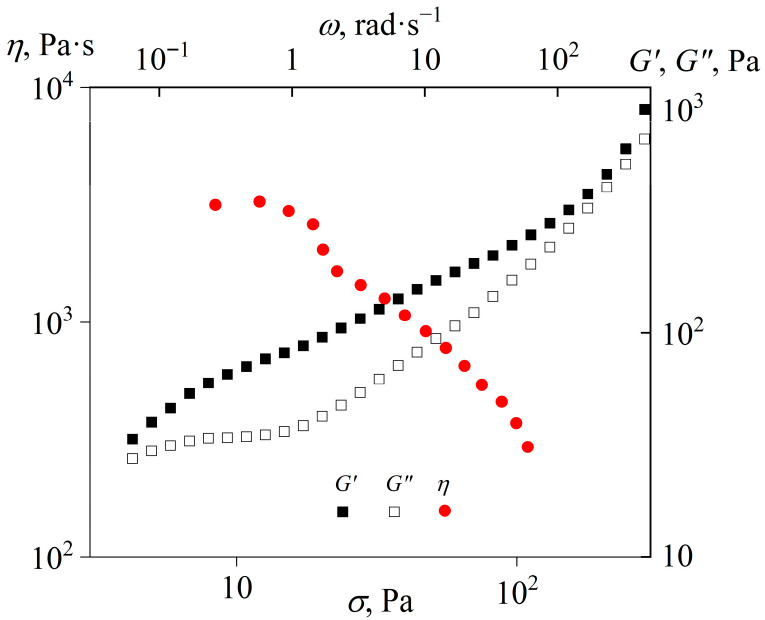
Dependencies of viscosity on the shear stress and storage and loss moduli on the angular frequency at 160 °C for the epoxy polymer cured with 40% [EMIM]Cl.

The viscosity and viscoelasticity of the cured epoxy polymer at an elevated temperature of 160 °C confirm its thermoplastic nature (Figure 11). The obtained polymer is flowable, having a low-shear viscosity of approximately 3000 Pa·s. An increase in the shear stress first reduces the viscosity, likely due to the polymer transitioning into a forced rubbery or glassy state [88], until the sample detaches from the measuring surface at shear stress above 100 Pa. The explanation comes from the fact that at high angular frequencies that correspond to high shear rates, the storage modulus of the resultant cured polymer exceeds its loss modulus. In turn, the predominance of elasticity in determining viscoelastic properties causes the accumulation of elastic deformation during continuous shear. This fact leads to the cessation of flow after some time from the start of deformation, followed by the rupture of the adhesive bond between the sample under study and the rheometer’s measurement surface. An increase in the angular frequency raises the storage and loss moduli, especially at high frequencies, which reflects the onset of mechanical glass transition.

In the case of the cured epoxy polymer containing 60% IL, its high-temperature studies were unavailable, as heating above 100–120 °C caused foaming, which likely results from the boiling of water absorbed from the air by the dispersed ionic liquid because of its high hygroscopicity.

Foaming at elevated temperatures is indicative of the substantial moisture ingress and the absence of a crosslinked network. To further verify this result, cured samples containing 40% and 60% [EMIM]Cl were subjected to solvent extraction in acetone at 25 °C for 72 h. Both materials dissolved completely, indicating the absence of a gel fraction and thereby confirming the formation of a linear or branched oligomeric system rather than a three-dimensional network. This conclusion is fully consistent with the rheological and dynamic mechanical analysis data, which show liquid-like behavior and a loss of dimensional stability upon heating.

**Figure 12:**
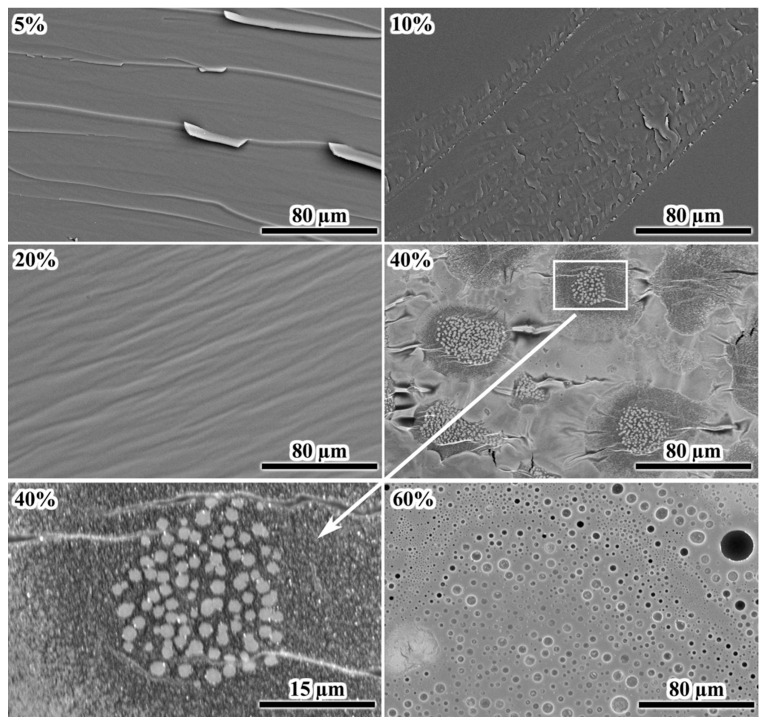
SEM images of the fracture surfaces of cured epoxy mixtures containing [EMIM]Cl, whose mass fractions are indicated in the upper left corners.

Examination of cross-sectional morphologies of the fractured samples after curing may give some additional information on their structures. At 5% IL, the fracture surface exhibits sharply protruding grooves, indicating brittle destruction (Figure 12). An increase in the IL content to 10–20% smoothes the grooves without forming a dispersed phase, suggesting that the excess IL, which has not reacted with the epoxy resin, remains dissolved within it. A dispersed phase appears at 40% IL content, forming clustered crystals of 1–2 µm. The epoxy matrix surrounding the clusters has a lower density due to its darker color. The density of epoxy polymers is lower than that of [EMIM]Cl (1.17–1.27 g/cm^3^ versus 1.435 g/cm^3^ [89,90,91]), indicating depletion of the epoxy matrix with the IL near its crystals, i.e., the IL crystallized because of phase separation and emission from the epoxy matrix. In other words, the IL exists in a dissolved metastable state in denser (brighter) regions of the crosslinked epoxy polymer, i.e., the IL would have formed a dispersed phase if not for the very high viscosity of the cured epoxy medium, which hinders crystallization.

**Figure 13:**
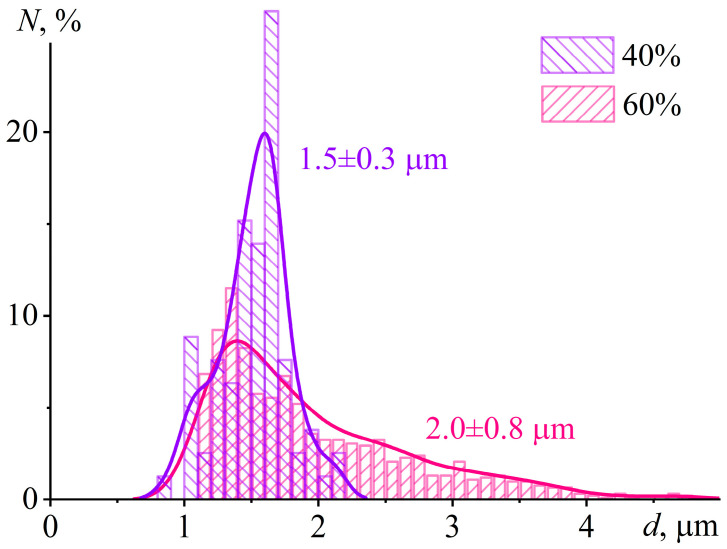
Number-weighted particle size distribution in the cured epoxy polymer containing 40 wt% or 60 wt% [EMIM]Cl. The number-average diameters are indicated near the curves.

At 60% IL content, the cured sample contains numerous droplets with sizes of 5–10 µm. Their spherical shape indicates their formation in a liquid epoxy matrix before it reaches high viscosity at a curing temperature of 160 °C. The sphericity and large size of the droplets contrast with the crystal formation at 40% IL content, where the small sizes and irregular shapes of the crystals suggest their formation under very high matrix viscosity. Interestingly, the particle size distribution at 40 wt% IL is unimodal and nearly symmetric, with a mean diameter of 1.5 ± 0.3 μm (Figure 13). In contrast, the distribution at 60 wt% IL is also unimodal but significantly broader (2.0 ± 0.8 μm), with a pronounced tail extending to 4 μm and beyond. This broader distribution suggests that IL droplets initially nucleate with sizes around 1–1.5 μm during cooling through the depressed melting point (≈60–70 °C). They subsequently undergo coalescence while the epoxy matrix remains in a liquid state (between 70 and 50 °C), where the viscosity is still low enough to permit droplet mobility and merging. Conversely, at 40 wt% IL, phase separation occurs much closer to the system’s glass transition (*T*_g_ = 65.2 °C). At this point, the matrix viscosity is already very high, which kinetically arrests droplet growth and effectively suppresses any coalescence, leading to the observed narrower size distribution.

Thus, the morphology observed at 40% [EMIM]Cl reflects a thermally induced phase separation arrested by the glass transition, rather than a reaction-induced phase separation. This distinction is critical because reaction-induced phase separation typically requires network formation and gelation to reduce miscibility and trigger demixing. However, the system does not gel at 40% IL (Figure 3， Figure 5b and Figure 11) and remains flowable, with viscosity peaking and subsequently decreasing during isothermal cure at 160 °C. The absence of a gel network indicates limited molecular weight growth and sparse crosslinking, consistent with epoxy group deficiency and the prevalence of chain termination or cyclization. Instead, phase separation occurs upon cooling. At the curing temperature of 160 °C, [EMIM]Cl is fully miscible with the epoxy resin. However, cooling introduces two factors that drive demixing: (1) decreased solubility of [EMIM]Cl in the epoxy matrix and (2) the approach of the temperature to the depressed melting point of the IL, which is approximately 60 °C as inferred from *T*_g_ = 65.2 °C (see Figure 9 and Table 2). In other words, crystallization initiates below 60 °C, but matrix viscosity rises sharply near *T*_g_, rapidly suppressing molecular diffusion and halting crystal growth. Consequently, the resulting morphology—small (1–2 µm), irregular, clustered crystals—is kinetically trapped rather than equilibrium-controlled. In contrast, the 60% IL sample undergoes phase separation earlier—at a higher temperature and lower viscosity—yielding spherical droplets of 5–10 µm characteristic of liquid–liquid demixing in a low-viscosity medium. This stark morphological difference between the 40% and 60% samples directly maps onto the transition from a thermally induced, glass-transition-arrested phase separation to an early-stage liquid–liquid separation. In the latter case, the high IL concentration pushes the system above the binodal even at elevated temperatures. Consequently, phase separation occurs during isothermal cure at 160 °C when viscosity is still relatively low, allowing capillary forces to minimize interfacial energy and form spherical domains. This judgement is further supported by the observation that heating the 60% sample above 100 °C leads to foaming, consistent with IL-rich domains retaining sufficient mobility to coalesce, absorb moisture, and undergo boiling.

### 2.4. A Concentration-Dependent Strategy: From Reinforcement to Functional Hybrids

In general, the concentration-dependent evolution of structure and properties in the DGEBA/[EMIM]Cl system aligns with and extends recent trends in the design of construction and functional epoxy materials.

The usual approach to increase the strength and stiffness of epoxy polymers is to modify them with filler nanoparticles, such as carbon nanotubes, MXenes, or organomodified montmorillonite, which work well even for improving composites reinforced with aramid [92,93] or carbon fibers [94,95]. At 5 wt% IL, the formation of a stiff network (*G*′ = 6.95 GPa, Table 2) demonstrates how molecular-scale incorporation of imidazolium fragments can also increase flexural modulus without the need for reinforcement with filler particles. This approach is conceptually similar to the development of bio-based benzoxazines containing phthalonitrile moieties, which, however, require complex synthesis to achieve superior properties [96]. In contrast, the present method employs a single-component, solvent-free IL as the curing agent, adhering to the principles of green chemistry.

At 10–20 wt% IL, the cured materials exhibit a reduced glass transition temperature and a lower flexural modulus, which is characteristic of internal plasticization by unreacted [EMIM]Cl. This observation aligns with the established role of imidazolium chlorides as dual-function modifiers in epoxy systems. For instance, it has been demonstrated that even low loadings (≈0.2 wt%) of 1-butyl-3-methylimidazolium chloride in a DGEBA/TETA system reduce the crosslink density while increasing fracture toughness by 25.4%, owing to enhanced chain mobility and the formation of secondary bonds [97]. In our system, the significantly higher IL content (10–20 wt%) shifts the balance toward more pronounced plasticization. Crucially, this occurs without macroscopic phase separation, indicating retained molecular compatibility. This behavior suggests a promising route for formulating high-toughness materials where enhanced ductility is prioritized over maximum rigidity. In some respects, this behavior also resembles the modification of epoxy resins with asphaltenes, where a content of 5–10 wt% leads to plasticization, reducing the glass transition temperature and flexural modulus [71]. However, at 20 wt%, asphaltenes act as a reinforcing filler due to their limited compatibility with the cured epoxy matrix. This reinforcing effect is not observed with [EMIM]Cl, as its hygroscopic nature promotes the formation of a low-crystalline or even liquid state, preventing effective reinforcement.

At 40 wt% IL, the shift to thermoplastic behavior (*η* ≈ 3000 Pa·s at 160 °C, absence of a rubbery plateau) resonates with emerging efforts to design reprocessable epoxy systems. For instance, imidazolium-catalyzed dynamic covalent networks based on transesterification enable reshaping [98] but require synthetic modification of the resin. Our system, in contrast, achieves thermoplasticity intrinsically through stoichiometric imbalance—providing a simpler, catalyst-free route to recyclable materials derived from thermoset precursors.

Finally, the 60 wt% IL system, though limited as a phase-change material by hygroscopicity, serves as a valuable model for IL-rich functional composites. It was recently shown that a hydrophobic IL (1-dodecyl-3-methylimidazolium dodecylbenzenesulfonate) enables stable, high-IL-content DGEBA/diethylenetriamine coatings that exhibit antimicrobial activity [99]. Separately, a moderate ionic conductivity (8.32 × 10^−4^ S/m) was reported for an epoxy polymer containing 50% 1-ethyl-3-methylimidazolium bis(trifluoromethylsulfonyl)imide, which increased approximately 30-fold with the addition of carbon nanofibers to bridge the IL domains [100]. In our case, the ionic conductivity with 60% [EMIM]Cl is very similar (1.1 × 10^−3^ S/m), suggesting it could likewise be enhanced by the addition of conductive rod-like particles to percolate the ionic pathways.

Thus, our results establish the essential concentration–property relationships for hydrophilic IL/epoxy blends, providing a critical baseline for the rational design of next-generation functional hybrids for phase-change, antimicrobial, or ion-conductive applications. Future strategies for performance enhancement may involve incorporating secondary modifiers. These could include nanofillers to reinforce or percolate IL-rich domains, hydrophobic ILs to mitigate moisture uptake, or hybrid curing systems (e.g., combining the IL with an anhydride or amine) to tailor reactivity and final network architecture. Such integrated approaches hold the potential to unlock stable, high-IL-content composites with precisely tuned stiffness, ionic conductivity, and phase-change functionality.

## 3. Conclusions

The study of the structure and physicochemical properties of an epoxy resin cured with different concentrations of 1-ethyl-3-methylimidazolium chloride ([EMIM]Cl) revealed the following:

- At a 5% content, [EMIM]Cl acts as a curing agent for the epoxy resin, potentially outperforming a usual aliphatic amine and resulting in a crosslinked polymer with higher stiffness, although comprehensive investigations into its tensile and impact strength are necessary to substantiate claims of superior performance.
- A higher [EMIM]Cl concentration of 10% crosslinks the epoxy resin, but the excess ionic liquid acts as a plasticizer, reducing the stiffness and glass transition temperature.
- Concentrations of [EMIM]Cl in the range of 20–40% promote the dominance of the initiation reaction, which forms numerous growing chains that prematurely terminate and cyclize, resulting in a thermoplastic rather than crosslinked epoxy polymer.
- At 60% [EMIM]Cl content, a dispersed phase forms but absorbs water from the air because of hygroscopicity. It foams at high temperatures, does not crystallize, and does not act as an effective phase-change agent.

An advantage of [EMIM]Cl as a curing agent is its non-volatility and, consequently, non-toxicity during the curing of the epoxy resin, even at high temperatures. In addition, the imidazole ring incorporation into the crosslinked polymer provides its high stiffness, reaching 6.95 GPa when using 5% [EMIM]Cl. A disadvantage of the ionic liquid is its extreme hygroscopicity. Although [EMIM]Cl possesses suitable phase-change properties, including a melting temperature near 88 °C and a high latent heat of fusion (105 J/g), the hygroscopicity precludes its use as an effective phase-change material in epoxy matrices. The absorbed water depresses the melting point, reduces crystallinity, and causes foaming upon heating—thereby rendering the composite unsuitable for thermal energy storage. This result highlights a critical limitation of hydrophilic ionic liquids in PCM applications and underscores the necessity of employing hydrophobic ILs (e.g., long-chain-substituted cations paired with bis(trifluoromethylsulfonyl)imide anions) for the future development of IL/epoxy-based PCMs.

## 4. Materials and Methods

### 4.1. Materials

Diglycidyl ether of bisphenol A (DER-330, Dow Chemical, Midland, MI, USA), containing 180.5 g/mol-eq of epoxy groups, was an epoxy resin to obtain a continuous polymer matrix. 1-Ethyl-3-methylimidazolium chloride ([EMIM]Cl, Sigma-Aldrich, Steinheim, Germany) was an ionic liquid added to the epoxy resin as a curing agent and a potential phase-change agent at mass fractions of 5, 10, 20, 40, and 60%. These blends were heated to 80 °C to melt the ionic liquid and mixed using a magnetic stirrer until homogenization. Some of the resulting mixtures were cooled to 25 °C for studying the rheological properties and curing kinetics, while their other portions were poured into molds and cured at 160 °C for 4 h to investigate thermophysical and mechanical properties.

### 4.2. Methods

Rheological studies were conducted using a rotational rheometer DHR-2 (TA Instruments, New Castle, DE, USA). Isothermal investigations of uncured epoxy resin/[EMIM]Cl mixtures were performed using a cone–plate setup with a plate diameter of 40 mm and an angle between the cone and plate of 2° at 25 °C. Frequency dependencies were measured at a strain amplitude of 0.1% within an angular frequency range of 0.0628–628 rad/s. Flow curves were obtained at the same temperature under stepwise increases in shear rate from 10^−3^ to 1000 s^−1^. To determine temperature dependencies of the mixtures’ viscosity, a plate–plate setup with plates having an 8 mm diameter was used. The heating rate was 2 °C/min within a 25–250 °C temperature range at a shear rate of 100 s^−1^. The same measuring setup and shear rate were used to study the viscosity change during isothermal curing at 120, 140, 160, and 180 °C. Rheological characteristics were calculated using standard equations with no more than 5% measurement error.

Dynamic mechanical analysis (DMA) was performed on the same rotational rheometer using cured samples shaped into rectangular beams with a length of 32 mm, a width of 12 mm, and a thickness of 4 mm. Studies were conducted using the three-point bending test with a dual cantilever clamp at a frequency of 1 Hz, a strain amplitude of 0.01%, and a heating rate of 5 °C/min within a temperature range of 25–150 °C. Measurement errors for dynamic moduli did not exceed 5%.

Differential scanning calorimetry of samples before and after curing was done on a calorimeter MDSC 2920 (TA Instruments, New Castle, DE, USA) under an argon atmosphere. For uncured samples, the heat effect of epoxy resin crosslinking was measured at a heating rate of 2 °C/min within a temperature range of 25–260 °C. For cured samples, the glass transition temperature of the epoxy polymer and the heat effects of melting and crystallization of the dispersed IL were estimated at a heating rate of 10 °C/min within a temperature range of 25–160 °C. During enthalpy measurements, the relative error did not exceed 5%, while the accuracy of determining the transition temperatures was ±0.2 °C. The decomposition of two-step heat releases into separate transitions for evaluating their temperatures and enthalpies was performed using the software OriginPro 9.5 (OriginLab Corp., Northampton, MA, USA).

FTIR spectra of the cured epoxy/[EMIM]Cl systems were recorded on a Bruker IFS 66 v/s spectrometer (Karlsruhe, Germany) using a germanium crystal in attenuated total reflectance (ATR) mode. Spectra were acquired over the range of 600–4000 cm^−1^ at a resolution of 2 cm^−1^, averaging 32 scans.

Microphotographs of uncured samples placed between two cover glasses were obtained using a digital camera with a 12-megapixel 1/1.7-inch sensor IMX226 (Sony, Tokyo, Japan) and a ×25 magnification lens. Scanning electron microscopy (SEM) of cured samples was carried out using a microscope Phenom XL G2 (Thermo Fisher Scientific, Eindhoven, the Netherlands) at an accelerating voltage of 15 kV and a pressure of 60 Pa. A 5-nm-thick layer of silver was deposited onto the fracture surfaces of the samples via ion-plasma sputtering using a device 108 Auto (Cressington Scientific Instruments, Watford, UK) to prevent the accumulation of negative charge during the capture of SEM images.

## Figures and Tables

**Table 1:** The compositions of epoxy mixtures, the thermophysical characteristics of their curing using different concentrations of [EMIM]Cl, and their resultant glass transition temperatures.

*w*_IL_, wt%	[EMIM]Cl/Epoxy Group, mol/mol	*T*_max,1_, °C	Δ*H*_1_, J/g	*T*_max,2_, °C	Δ*H*_2_, J/g	Δ*H*_1+2_, J/g	*T*_g_, °C
5	1/15.4	140.0	99.3	210.6	260.4	359.3	83.3
10	1/7.31	148.6	223.3	207.5	205.6	428.9	81.3
20	1/3.25	148.2	333.9	191.9	158.4	492.3	66.7
40	1/1.22	149.7	257.1	194.0	172.8	429.9	51.6
60	1/0.54	155.5	327.5	–	–	327.5	48.8

**Table 2:** Glass transition temperature and flexural storage modulus of epoxy mixtures cured with [EMIM]Cl or other usual hardeners.

*w*_IL_, wt%	*T*_g,tan*δ*_, °C	*G’*_25°C_, MPa	*G’*_120°C_, MPa
5	102.8	6950	16.1
10	99.6	1160	1.5
20	84.4	620.1	<0.01
40	65.2	8.8	<0.01
11% TETA	109.9	1100	21.0
25.6% DDS	175.3	2960	–
48.3% MTHPA	64.2	1060	–

## Data Availability

The data presented in this study are available upon request from the corresponding author.
